# 

**Published:** 1873-05

**Authors:** 


### CONTENTS:

### ORIGINAL COMMUNICATIONS.

ARTICLE I.—Class Address. By J. B. Ten Eyck, M. D., D. D. S. .......... [illegible]

ARTICLE II.—The Brunonian or Molecular Movement. By S. P. Cutler, M. D., D. D. S. .......... [illegible]

ARTICLE III.—The Predisposing Causes of Dental Caries. By J. B. Ten Eyck, M D., D.D.S. 12

Dental Associations :—

Southern Dental Association .......... 16

American Dental Association .......... 16

California State Dental Association .......... 16

### SELECTED ARTICLES.

ARTICLE IV.—Irregularity. By Isaiah Forbes, D. D. S .......... 17
ARTICLE V.—Adhesion of the Soft Palate and Uvula to the Posterior Wall of the Pharynx —Operation—Cure. By A. B. Cooke, M. D .......... 25
ARTICLE VI.—The Alumni Associations and their Influence for Good .......... 29
ARTICLE VII.—The Best Method for Removing the Upper Maxillary Bone. By Julian J. Chisholm, M. D .......... 32
ARTICLE VIII.—Physiological Effects of Oxygen .......... 34

### EDITORIAL DEPARTMENT.

Decision of the Supreme Court of the United States in the Rubber Case .......... 36
Rubber Dam and its Application .......... 43
Dr. Arthur's Instrument .......... 45

### MONTHLY SUMMARY.

Application for Chilblains .......... 46

Chromic Acid .......... 46

Tea *vs.* Brandy .......... 46

Carbolic Acid as a Local Anæsthetic in Boils, Whitlows, &c .......... 47

Danger of Morphine and Chloroform .......... 47

Singular Causes of Death .......... 47

Anodyne Colloid .......... 48

Hops as an External Preparation .......... 48

